# Impact of Maternal Age on Oocyte and Embryo Competence

**DOI:** 10.3389/fendo.2018.00327

**Published:** 2018-06-29

**Authors:** Danilo Cimadomo, Gemma Fabozzi, Alberto Vaiarelli, Nicolò Ubaldi, Filippo Maria Ubaldi, Laura Rienzi

**Affiliations:** ^1^Clinica Valle Giulia, G.en.e.r.a. Centers for Reproductive Medicine, Rome, Italy; ^2^Catholic University of the Sacred Heart, Rome, Italy

**Keywords:** ovarian reserve, oocyte competence, aging, aneuploidies, IVF

## Abstract

The overall success of human reproduction, either spontaneously or after IVF, is highly dependent upon maternal age. The main reasons for age-related infertility include reduced ovarian reserve and decreased oocyte/embryo competence due to aging insults, especially concerning an increased incidence of aneuploidies and possibly decreased mitochondrial activity. Age-related chromosomal abnormalities mainly arise because of meiotic impairments during oogenesis, following flawed chromosome segregation patterns such as non-disjunction, premature separation of sister chromatids, or the recent reverse segregation. In this review, we briefly discuss the main mechanisms putatively impaired by aging in the oocytes and the deriving embryos. We also report the main strategies proposed to improve the management of advanced maternal age women in IVF: fertility preservation through oocyte cryopreservation to prevent aging; optimization of the ovarian stimulation and enhancement of embryo selection to limit its effects; and oocyte donation to circumvent its consequences.

## Introduction

**Figure 1:**
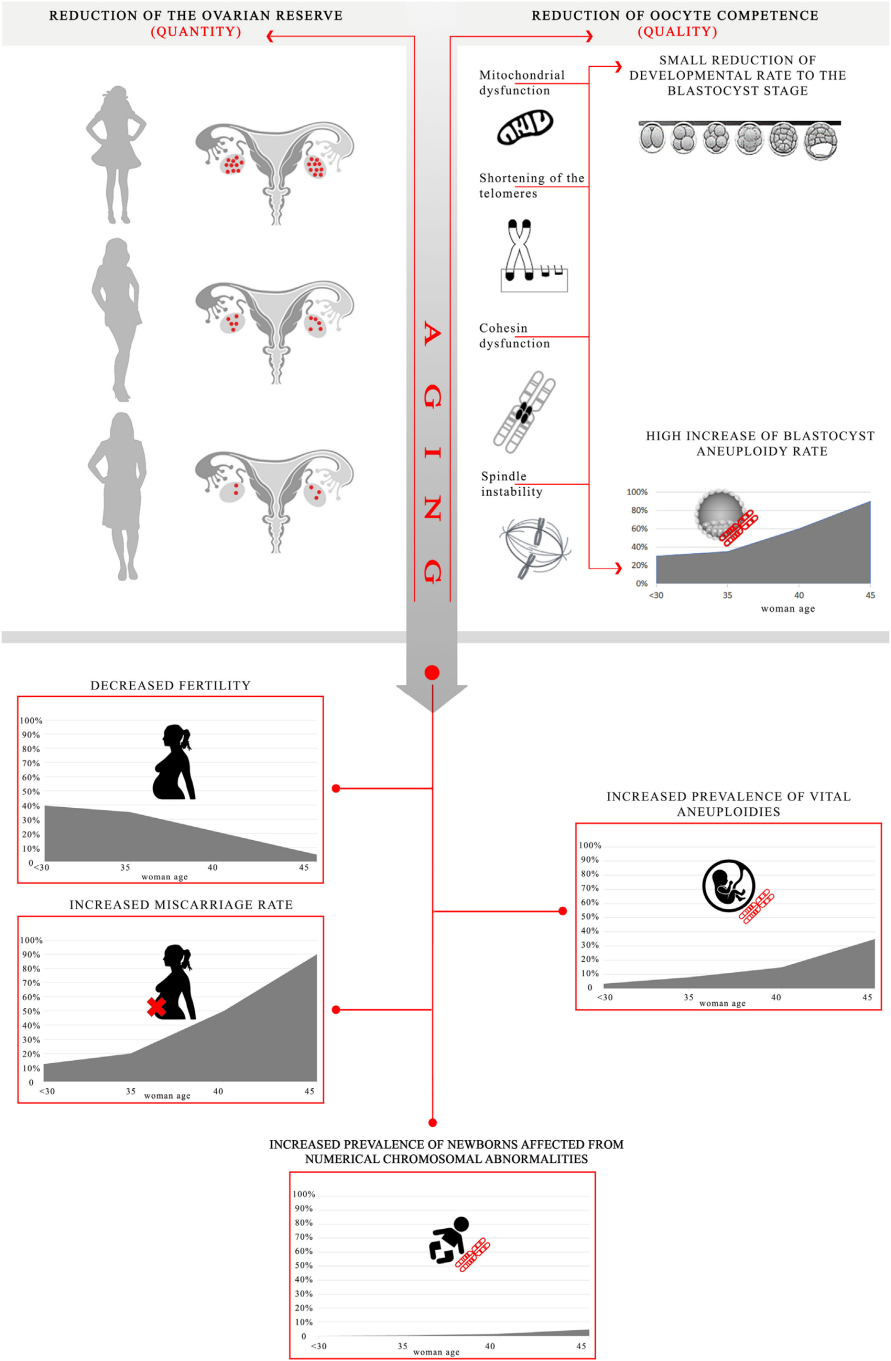
Effect of advanced maternal age on oocyte/embryo competence and putative mechanisms impaired by aging. Aging in women causes both a reduction of the ovarian reserve and of the oocyte competence. All the processes impaired may result into a lower energy production/balance involving a small reduction of embryo developmental rate to the blastocyst stage, as well as a higher frequency of chromosome missegregation during maternal meiosis leading to a high increase in blastocyst aneuploidy rate (especially in women older than 35) [*data adapted from Franasiak et al*. (10) and *Capalbo et al*. (11)]. Ultimately, these mechanisms converge into a decreased fertility, an increased prevalence of vital chromosomal abnormalities, an increased miscarriage rate, as well as an increased prevalence of numerical chromosomal abnormalities in the newborns [*data adapted from Hassold and Hunt* (13) and *Heffner* (4)]. *The aneuploidy rate is estimated per biopsied blastocyst; the fertility is estimated as number of babies born per 1,000 married women; the overall prevalence of vital aneuploidies is estimated per clinically recognized pregnancy; the miscarriage rate is estimated per clinical pregnancy; at last, the overall prevalence of numerical chromosomal abnormalities is estimated per number of newborns*.

Human reproduction success is highly dependent upon the age at which women attempt to conceive, which is progressively increasing worldwide (1, 2). Fertility decreases as the woman ages, while the incidence of miscarriage and the prevalence of vital chromosomal abnormalities follow an opposite trend (2–4) (Figure 1). In IVF, maternal age is among the strongest predictors of success (5). Specifically, advanced maternal age (AMA; defined as ≥35 years) shows just a negligible impact upon fertilization rate (6, 7) and a mild impact upon embryo development to the blastocyst stage (8, 9), but results in a dramatic impact upon blastocyst aneuploidy rate (10, 11) (Figure 1). However, the molecular and biochemical mechanisms involved in age-related infertility and their impact on oocyte and embryo quality remain to be clearly elucidated. Up to date, several dysfunctions have been associated with impaired fertility in aged women. Together with a progressive reduction of the ovarian reserve, woman aging involves also a compromised competence of the oocytes/embryos because of defective physiological pathways, such as energy production and balance, metabolism, epigenetic regulation, cell cycle checkpoints, and increased meiotic missegregation (11, 12). In this review, we provide a summary of the main putative causes for the age-related decrease in oocyte/embryo competence, along with the mechanisms underlying aging and the main clinical strategies proposed to prevent/limit the impact of AMA upon IVF success.

## Maternal Aging and Aneuploidies

The oocyte must sustain embryo development until embryonic genome activation (EGA) (14). To effectively reach EGA, synchronous nuclear and cytoplasmic maturation are required. Any failure in these processes may cause an incorrect transition from a maternal to an embryonic control upon embryo development. However, after birth and until follicle recruitment and ovulation, the oocytes enter a protracted arrest in the prophase of meiosis I, during which they are subject to the detrimental effects of aging, especially impairing the genetic stability (15), and ultimately affecting the chance of success in human reproduction. Indeed, the oocytes hold most of the reproductive potential in humans, as demonstrated by the restored fertility in women who undergo egg donation (16).

Maternal age is the main cause of embryonic aneuploidies (4, 13, 17). More than 90% of these imbalances are indeed of maternal origin caused by chromosomal missegregation during oogenesis (15). Mainly meiosis I errors may occur (>70% of cases), which although can be “corrected” in meiosis II, thereby resolving the initial error (11).

If full-chromosome constitutive aneuploidies are mainly generated by a defective maternal meiosis, structural chromosomal abnormalities (e.g., balanced translocations) seem instead independent from maternal age and may equally affect both the partners together with segmental aneuploidies, copy number variations, microdeletions/microduplications, and post-zygotic mitotic errors. Indeed, they probably arise from *de novo* events during either oogenesis and spermatogenesis or mitosis (18, 19).

The maintenance of the bivalent structure is a critical issue in maternal meiosis. In humans, homologous chromosomes recombine in primary oocytes during fetal development to form a bivalent configuration at meiotic prophase I. This configuration must be maintained for years, along which the oocytes remain arrested at the G2/M transition (dictyate stage) until menarche. At this stage, meiosis resumption and chromosome segregation take place. However, during this extended period of quiescence, the bivalent structure may weaken, leading to the formation of univalents or to sister chromatids splitting at meiosis I. The incidence of both these events indeed correlates with increased maternal age and reduced recombination rate (20–25), but the related causative mechanisms are still unclear. Two hypotheses have been proposed: (i) the univalents originate from bivalents deterioration throughout the dictyate arrest or (ii) the oocytes that underwent deficient recombination are ovulated last from the ovary.

Surprisingly, Ottolini and colleagues recently reported, *via* the karyomapping technique (a method that through the specific parental haplotypes allows the definition of an SNPs-based map of each chromatid) applied to artificially activated human oocytes and their polar bodies, that the most common non-canonical segregation pattern is reverse segregation (26). According to this novel segregation scheme, which cannot be identified by conventional copy number analysis, the non-sister chromatids, instead of the homologous, segregate together in meiosis I. This pattern, even if unconventional, does not result in an unbalanced chromosomal constitution *per se*, unless it is followed by a further error during meiosis II. Therefore, the most common segregation error of maternal meiosis reported in the majority of activated/fertilized oocytes is still the premature separation of sister chromatids (PSSC) in meiosis I (27–30). At last, meiosis I or meiosis II non-disjunction events should be accounted as causes of maternal meiotic impairments, even though probably less frequent than what previously reported (31, 32).

Hereafter, we summarize the molecular and cellular processes that may be affected because of aging in the oocytes (33, 34): mitochondrial dysfunction, shortening of telomeres, cohesins dysfunction, and meiotic spindle abnormalities due to spindle-assembly checkpoint (SAC) impairment. Reduced development to the blastocyst stage and/or chromosomal abnormalities are their putative consequences (Figure 1).

## Putative Mechanisms Impaired by Aging and Leading to a Reduced Oocyte/Embryo Competence

### Mitochondrial Dysfunction

Mitochondria are the most numerous organelles in the oocyte and represent its powerhouse. They are characterized by their own genome (mtDNA) and constitute the main maternal contribution to embryogenesis (35). Indeed, the sperm does not provide mitochondria to the offspring. They are considered pivotal especially in the delicate first phases of preimplantation development, when a balanced energy consumption is crucial for an efficient oocyte cytoplasmic and nuclear maturation, throughout processes such as germinal vesicle breakdown, or microtubule assembly and disassembly during meiotic spindle formation (36, 37). Moreover, mitochondria cover an essential role in various signaling pathways, such as Ca^2+^ signaling and regulation of the intracellular red-ox potential, particularly important for fertilization and early development (36, 38).

The adverse effect of aging upon the mitochondria within the oocyte has been widely reported: mitochondrial swelling, vacuolization, and cristae alteration have been described as common structural features of oocytes from AMA patients (39, 40). For instance, the mitochondrial membrane potential, which mirrors mitochondrial activity, is progressively altered (41). Similarly, a reduced ATP production and decreased metabolic activity in aged oocytes has been highlighted, which in turn may contribute to impairments in meiotic spindle assembly, cell cycle regulation, chromosome segregation, embryo development, and finally implantation (40, 42).

Mitochondrial-DNA lacks protective histones and efficient DNA repair mechanisms. Therefore, mtDNA mutation rate is about 25-times higher than nuclear-DNA one (43). Clearly, the longer the quiescent period, the higher the risk for mtDNA errors. Furthermore, also the overall concentration of mtDNA seems to be decreased in the oocytes from older patients (44, 45), thereby concurring to a lower oocyte/embryo competence (46–48). Of note, in humans, mitochondrial biogenesis is physiologically activated only at the blastocyst stage (40, 49) to limit the oxidative phosphorylation-induced stress in the first phases of embryo development. In older patients, the reduced amount and/or faulty activity of the pre-existing mitochondria within the oocyte may induce a compensatory premature initiation of mitochondrial biogenesis (50), which in turn may contribute to early embryo developmental failure (48).

Recently, mtDNA content in trophectoderm biopsies at the blastocyst stage has been proposed as a putative biomarker of implantation potential. However, the clinical studies conducted to date reported controversial results (48, 51–54). Indeed, lately, Humaidan and colleagues warned that it is still difficult to discriminate between “fact and fiction” in the current scenario and mtDNA cannot be considered a new biomarker of embryonic implantation potential (55): extensive validation, as well as more pre-clinical and possibly non-selection data, are yet required. Until then, the quantification of mtDNA from trophectoderm biopsies should be considered still an experimental procedure.

The mitochondria are also present in the granulosa cells (GCs) surrounding the oocyte already in the early phases of oogenesis. GCs are directly involved in establishing oocyte competence during oogenesis thanks to the well-known bi-directional dialog between these two sections of the follicle (56, 57). As for the oocytes, also GCs from AMA women showed higher levels of mtDNA deletions (58) and damaged mitochondria (59). The amount of mtDNA in GCs has been also reported to correlate with embryo quality (60) and poor ovarian reserve. The current hypothesis is that as the mtDNA in the oocyte supports the early embryonic development, similarly the mtDNA on its related GCs supports oocyte maturation, both possibly modulating embryo competence. Such hypothesis is supported by the high correlation between the mtDNA levels in the two compartments of the follicle (61).

In summary, aging can compromise both mtDNA integrity and/or mitochondria morphology or alter the microenvironment within the follicle and perturbate the mutual crosstalk between the oocyte and its GCs (39, 40, 62).

### Shortening of the Telomeres

The telomeres are short tandem repeats of specialized-DNA sequences that protect chromosome ends (63). Their function is essential for meiosis since, during the early prophase, the telomeres tether the chromosomes to the nuclear membrane to facilitate homologous pairing and initiate synapsis to form chiasmata, the physical sites of recombination responsible for normal segregation, thereby preventing non-disjunction (64, 65). Age-related telomeres shortening occurs either in dividing or non-dividing cells and has been associated with several age-related diseases (e.g., diabetes, cardiovascular diseases, and cancer) (66, 67). However, telomere dynamics extensively differ according to the cell type and gender. For instance, in the male germline, the length of the telomeres is preserved with aging, probably due to a constant activity of the telomerase (the reverse transcriptase involved in telomeres extension), which is expressed at high levels in the spermatogonia (68). Interestingly, an even increased mean length of the telomeres, as well as a higher length heterogeneity, has been recently reported in aged men with respect to younger patients (69). Conversely, the telomeres in the oocytes begin shortening during fetal oogenesis, and this process is continued in the adult ovary, probably due to the chronic effects of oxidative and genotoxic stress, the late exit of the female gametes from their cell cycle arrest, as well as to a reduced activity of the telomerase (68, 70, 71). Furthermore, it has been demonstrated that the telomeres are shorter in oocytes from women who experienced IVF failure or recurrent miscarriage (72), as well as in oocytes resulting in fragmented (73) or aneuploid embryos (74). To this regard, Keefe and colleagues postulated the evolutionistic “telomere-mediated oocyte aging” theory: preventing AMA women from conceiving would, in turn, prevent them from dying because of childbirth, thereby affecting the reproductive fitness of their offspring (70, 75).

### Cohesin Dysfunctions

Loss of cohesion between sister chromatids close to the centromeres is another age-related dysfunction which may cause chromosomal missegregation. Cohesins are a complex of proteins that holds sister chromatids together after DNA replication and is responsible for maintaining the bivalent structure throughout the extended period of quiescence. Only at anaphase, the cohesins are removed to trigger the separation of sister chromatids. Gathering evidence is outlining an age-related disruption of cohesin function leading to missegregation within the oocyte, especially in the presence of low recombination rate (76). For instance, cytogenetic studies of human oocytes and embryos showed that PSSC is often associated with the age-related reduction of cohesins (e.g., Rec8, SA3, and SMC1b) (77, 78). Furthermore, also the activity of the regulatory proteins preventing a precocious removal of the cohesins seems to decline in an age-related fashion (79), regardless their nuclear location, which theoretically should protect them from the insults of mechanical stress and/or reactive-oxygen-species. Finally, a structural and functional interaction exists between cohesins and telomeres in mice (80). Therefore, in AMA patients, the age-related issues that affect the telomeres may trigger similar dysfunctions in the cohesins’ activity (76).

### Spindle Instability

The meiotic spindle is responsible for the separation of both homologous chromosomes and sister chromatids, therefore essential to ensure an accurate segregation (81). Aberrations in its assembly seem to contribute to the higher prevalence of aneuploidies in older women (82). These aberrations may also be ascribed to a decreased metabolic activity of mitochondria, resulting into a reduced amount of ATP because of AMA. The spindle of young oocytes is compact, orthogonally oriented with respect to the oolemma and each pole is associated with a ring of centrosome proteins. Conversely, nearly 80% of the oocytes in AMA patients may exhibit abnormal spindles with an elongated and/or smaller profile and few microtubular foci at the cortex (81, 82). To this regard, also the SAC, a ubiquitous safety protein complex that ensures a correct spindle formation (83), shows a reduced stringency with AMA (84–86). Different protein components of SAC (e.g., Mad2 and Bub1) showed indeed lower concentrations in oocytes from older women (84, 87).

### Other Putative Mechanisms Impaired by Aging

Gene expression studies in oocytes from several species indicate that the activity of gene products involved in cell cycle regulation, spindle formation, and organelle integrity may be altered in oocytes from older individuals. For instance, in both murine and human oocytes ~5% of all the transcripts detected at the MII stage were found to be affected by aging (88, 89). Possibly, the divergent signatures derive from the altered patterns of epigenetic modifications (e.g., methylation and acetylation), which have been indeed reported in both species (90–94). This field of reproductive genetics requires extensive investigations in the next years to better unveil these mechanisms.

## Clinical Considerations

A clear correlation exists between increasing maternal age and decreasing success in conceiving both spontaneously and after IVF (4, 5). Both reduced ovarian reserve and oocyte quality contribute to this scenario. Currently, no therapy exists to counteract infertility in AMA patients and we can only try to limit this biological and social issue.

First, fertility preservation *via* oocyte cryopreservation (95, 96) provides a valuable option to all women (not only oncological patients) aiming to prevent the natural decline of oocyte competence. Yet, the age at which fertility preservation is performed is an important effector of the ultimate outcome (<35 years is preferable), and obviously the pregnancy cannot be guaranteed by oocyte banking (97).

Second, the maximization of ovarian reserve exploitation through tailored controlled-ovarian-stimulation (COS) is crucial to increase the number of oocytes collected, thereby also increasing the chance of success after IVF (98, 99). A higher number of oocytes collected per ovarian cycle might indeed compensate for the decrease in both oocyte quantity (i.e., ovarian reserve) and quality (i.e., competence). Therefore, novel COS strategies, such as oocyte/embryo accumulation in consecutive cycles (100) or double ovarian stimulation in the same ovarian cycle [i.e., the Shanghai (101) or the DuoStim protocol (102)], have been recently proposed to shorten the time invested by poor prognosis patients in their pursuit of a live birth. Promising data have been reported to this regard, especially in terms of cost-effectiveness and safety.

Third, the enhancement of embryo selection *via* preimplantation-genetic-testing represents another important option in AMA patients. In fact, the goal of ART is to achieve the birth of a healthy child minimizing the risks for the patient, and this is particularly true in AMA when the incidence of aneuploidies dramatically increases (10). This approach, by avoiding the transfer of aneuploid blastocysts and their related risks (i.e., implantation failures, miscarriages, and affected child), might result in an increased efficiency of each IVF treatment (103, 104). Importantly, once an euploid blastocyst is identified, its implantation potential is independent of maternal age (45–50%), thereby allowing the adoption of a single-embryo-transfer policy also in AMA patients, concurrently lowering the risk for multiple gestations and their related obstetrical/perinatal risks (105, 106). Soon, the implementation of -omic sciences and the pursuit of non-invasiveness and higher cost-effectiveness in this field may converge and bring about intriguing avant-gardes to further improve embryo selection.

Finally, oocyte donation represents an effective approach to circumvent the age-related fertility decline. Recently, the optimization of cryopreservation techniques and the constitution of oocyte-banking facilities and programs allowed us to avoid synchronization between donors and recipients. Indeed, similar success rates derive from either fresh or frozen oocytes (107). Yet, in some countries oocyte donation is still forbidden and ethical/psychological concerns limit its large-scale adoption.

## Conclusion

Currently in IVF, a panel of experts focused on the management of poor prognosis patients, known as the POSEIDON group (Patient-Oriented Strategies Encompassing IndividualizeD Oocyte Number), has redefined the aim of ovarian stimulation (108). Specifically, they claimed that COS should be tailored “to retrieve the number of oocytes needed for the specific patient to obtain at least one euploid embryo for transfer.” Such statement is based on two important assumptions: (i) aneuploidy rate in human blastocysts increases from a 30% baseline in women younger than 35 to >90% in women older than 44 (10, 11) and (ii) the number of eggs collected and embryos obtained during IVF does not alter this rate (109). In other terms, the definition of the number of oocytes required (quantity) from each patient should entail the estimate of their competence (quality) aiming at obtaining at least one euploid blastocyst. Then, when performed, a euploid blastocyst transfer results into a healthy live birth in ~50% of cases, regardless woman age (103).

To conclude, evidence-based data should always guide the counseling and the patients should be scrupulously informed about their estimated chance to conceive, especially if older than 35. Indeed, 35 years should be the lowest age-threshold to define AMA and 45 years should be considered the highest age-threshold to undergo IVF with own eggs, at least according to the latest published report (9).

## Author Contributions

DC and GF drafted the manuscript. All authors contributed in the literature search and discussion of the published evidence.

## Conflict of Interest Statement

The authors declare that the research was conducted in the absence of any commercial or financial relationships that could be construed as a potential conflict of interest.
